# Orientation-Induced Structure–Property Relationships in Recycled PET-Based Blends Containing Amorphous Copolyesters and Polycarbonate

**DOI:** 10.3390/polym18111293

**Published:** 2026-05-25

**Authors:** Nadiya Sova, Bogdan Savchenko, Aleksander Slieptsov, Viktoriia Plavan, Alina Vozniak, Victor Beloshenko

**Affiliations:** 1 Kyiv National University of Technology and Design, Nemirovicha Danchenko Street, 2, 01011 Kyiv, Ukraine; djanc@ukr.net (N.S.); 1079@ukr.net (B.S.); zikmne@gmail.com (A.S.); plavan.vp@knutd.edu.ua (V.P.); 2 Centre of Molecular and Macromolecular Studies, Polish Academy of Sciences, Sienkiewicza Street, 112, 90001 Lodz, Poland; 3 Donetsk Institute for Physics and Engineering Named After O.O. Galkin, National Academy of Sciences of Ukraine, Pr. Nauki, 46, 03028 Kyiv, Ukraine; biloshenko.va@gmail.com

**Keywords:** polyethylene terephthalate, polyethylene terephthalate glycol-modified, polycyclohexylenedimethylene terephthalate glycol-modified, polycarbonate, orientation drawing, polymer blends, recycling

## Abstract

The presence of polymeric impurities in recycled polyethylene terephthalate (PET) streams, particularly amorphous copolyesters such as polyethylene terephthalate glycol-modified (PETG) and polycyclohexylenedimethylene terephthalate glycol-modified (PCTG), as well as polycarbonate (PC), represents a critical challenge for high-performance applications involving orientation drawing. In this study, the influence of such components on the orientation behavior and resulting mechanical properties of PET-based blends was systematically investigated. Model blends were prepared using virgin materials and processed into monofilaments via inline melt spinning followed by controlled orientation drawing with draw ratios up to 6.5. The evolution of tensile strength, modulus, elongation at break, density, and thermal shrinkage was analyzed as a function of draw ratio. The results demonstrate that PET-rich systems exhibit superior mechanical performance, which is consistent with the development of strain-induced crystallization during orientation drawing, reflected in increased density and reduced thermal shrinkage at higher draw ratios. In contrast, amorphous copolyesters (PETG, PCTG) suppress crystallization, resulting in limited structural development, lower modulus, and significantly higher thermal shrinkage. Blends containing polycarbonate showed reduced maximum draw ratios and tensile strength, indicating restricted orientation capability, but exhibited comparatively low shrinkage at moderate draw levels. The study establishes clear structure–property relationships linking molecular orientation, crystallization behavior, and macroscopic performance in PET-based blends. The findings highlight that even minor amounts (~10 wt%) of amorphous polyester impurities can significantly alter orientation efficiency and end-use properties, emphasizing the importance of feedstock control in recycling processes targeting high-performance-oriented products such as strapping tapes and monofilaments.

## 1. Introduction

Recycled polymers are increasingly used in modern industry due to economic and environmental considerations [1,2,3,4,5]. However, many high-value applications impose strict requirements on mechanical performance and long-term dimensional stability, making the quality and composition of recycled feedstock critically important. One of the key challenges in polymer recycling is the effective separation of materials with similar physical appearance and processing behavior, particularly within the polyester family [6,7,8].

Polyethylene terephthalate (PET) is among the most widely recycled polymers worldwide and is commonly used in the form of copolymers rather than pure homopolymers [9]. The incorporation of comonomers such as isophthalic acid or cyclohexanedimethanol is primarily aimed at modifying crystallization behavior, reducing crystallization rate, and improving optical clarity in packaging applications [10]. As a result, modern polyester waste streams increasingly contain not only semi-crystalline PET but also amorphous copolyesters such as polyethylene terephthalate glycol-modified (PETG) and polycyclohexylenedimethylene terephthalate glycol-modified (PCTG), as well as other transparent polymers including polycarbonate (PC). Among such contaminants, PETG, PCTG, and polycarbonate (PC) are particularly relevant because they are widely used in transparent packaging, thermoformed products, technical parts, and consumer goods, and are therefore frequently present in mixed post-consumer PET waste streams. In addition, these materials possess similar density and processing behavior to PET, which makes separation by conventional flotation-based sorting methods extremely difficult in industrial recycling systems. The increasing use of PETG and PCTG in thermoforming and additive manufacturing applications further contributes to their growing presence in recycled PET waste streams. Due to their similar optical appearance and overlapping processing windows, efficient separation during industrial recycling remains challenging [6,7,8].

In many recycling applications, particularly those involving fibers, monofilaments, and strapping tapes, orientation drawing is employed to enhance mechanical properties. This process induces molecular alignment under tensile deformation and, in semi-crystalline polymers, may be accompanied by strain-induced crystallization. The combined effect of chain alignment and crystallization plays a decisive role in determining the final mechanical performance, stiffness, and thermal stability of oriented products [11,12].

For semi-crystalline polymers such as PET, orientation drawing can significantly improve tensile strength and modulus through lamellar reorganization and strain-induced crystallization, which stabilize the oriented structure. In contrast, amorphous polymers such as PETG and PCTG exhibit limited ability to crystallize, and their deformation is primarily governed by chain alignment within the amorphous phase. As a result, structural development during drawing may be restricted, affecting the resulting properties. When such materials are present in polymer blends, the situation becomes more complex due to possible incompatibility, phase separation, and non-cooperative deformation mechanisms [13,14,15,16,17].

Although the general properties of polyester-based blends have been widely studied [18,19,20,21,22], their behavior under orientation drawing remains insufficiently understood. In particular, there is a lack of systematic studies addressing how small amounts of amorphous polyester impurities or polycarbonate influence orientation efficiency, drawability, and the resulting structure–property relationships in PET-based systems [23,24].

Previous studies have investigated crystallization behavior, miscibility, and mechanical performance of PET-based blends containing amorphous copolyesters and polycarbonate [15,19,20,23]. Most of these works focused on injection-molded materials, films, or additive manufacturing systems, where structural development occurs predominantly under quiescent or weakly oriented conditions. In contrast, the behavior of such blends during uniaxial orientation drawing remains insufficiently understood, particularly at impurity levels relevant to industrial recycling streams. In practical recycling systems, even relatively small amounts of amorphous contaminants may significantly affect orientation efficiency, strain-induced crystallization, dimensional stability, and mechanical performance of oriented products such as monofilaments and strapping tapes.

This issue is especially relevant in real recycling conditions, where feedstock is often derived from mixed post-consumer waste and cannot be fully separated by polymer type. Advanced sorting technologies such as near-infrared (NIR) spectroscopy are not always available due to economic constraints, leading to the presence of minor fractions of PETG, PCTG, and PC in recycled PET streams.

The present study aims to investigate the influence of such impurities on the orientation drawing behavior of PET copolymer-based blends. Model systems with controlled compositions of PET, PETG, PCTG, and polycarbonate were prepared and processed into monofilaments, followed by uniaxial orientation drawing. The evolution of mechanical properties, density, and thermal shrinkage as a function of draw ratio was analyzed to establish relationships between molecular orientation, crystallization behavior, and macroscopic performance.

The results provide insight into the role of amorphous and incompatible components in limiting orientation efficiency and highlight the importance of feedstock control for high-performance applications of recycled PET, such as strapping tapes and technical monofilaments.

In the context of the transition towards a circular economy, mechanical recycling of PET via melt processing and orientation drawing remains the most widely implemented and economically viable approach [25,26,27]. Under these conditions, even minor compositional variations can significantly influence structural development during processing and the resulting material performance.

## 2. Materials and Methods

### 2.1. Materials and Blend Preparation

The following polymers were used in this study: a general-purpose bottle-grade PET copolymer (SKYPET BR, SK Chemicals, Seongnam, Republic of Korea), PETG (Eastar™ GN101, Eastman Chemical Company, Kingsport, TN, USA), polycarbonate (Makrolon™ ET3137, Bayer AG, Leverkusen, Germany), and PCTG (SkyGreen™ JN200, Sky Chemicals).

Model blends were prepared to simulate typical contamination scenarios encountered in recycled PET streams. The compositions of the investigated systems are summarized in Table 1. In addition to the neat materials, binary blends containing 10 wt% of a secondary component were selected to represent realistic impurity levels. The 10 wt% impurity level was selected as a model composition representing poorly sorted recycled PET streams or mixed-polymer waste fractions. Although lower impurity concentrations (1–5 wt%) are expected to produce qualitatively similar effects, their influence on crystallization suppression, drawability, and mechanical performance would likely be less pronounced. The selected concentration therefore allows clearer visualization of the mechanisms governing structural disruption and orientation-induced property changes.

Prior to processing, all materials were dried in a hot air oven (Memmert GmbH, Schwabach, Germany) according to the manufacturers’ recommendations. Due to the higher drying temperature required for PET (150 °C), it was dried separately, cooled to 75 °C, and subsequently mixed with the other components. The resulting blends were then dried at 75 °C before extrusion.

Melt compounding was carried out using a custom-built laboratory single-screw extruder (Kyiv National University of Technology and Design, Kyiv, Ukraine) at a temperature profile of 240–275–275–265 °C. The extruded filament obtained during the mixing stage was pelletized and re-dried prior to further processing. This two-step processing route was employed to simulate industrial recycling conditions involving regranulation and reprocessing.

### 2.2. Filament Extrusion and Orientation Drawing

**Figure 1:**
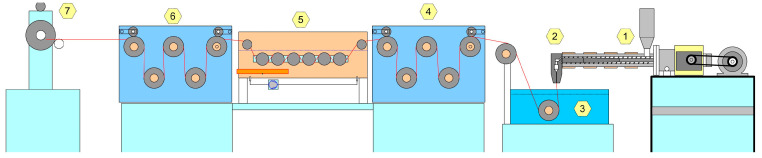
Schematic representation of the laboratory-scale extrusion and drawing setup: 1—extruder; 2—die head; 3—cooling bath; 4,6—five-roll drawing unit; 5—drawing water bath; 7—winder.

Monofilaments were produced using a laboratory-scale extrusion and drawing setup (Figure 1), consisting of a single-screw extruder, a die head, a cooling bath, a two-stage five-roll drawing unit, a hot water drawing bath, and a winding system.

Extrusion was performed using a single-screw extruder (D = 27 mm, L/D = 28, compression ratio 3.0) at a temperature profile of 240–275–260–260 °C, with a melt temperature of approximately 265 °C. The blends were extruded vertically through a round die (diameter 3.75 mm, L/D = 14). The as-extruded filament (diameter ~3.5 mm) was obtained at minimal take-up speed and subsequently cooled in a water bath maintained at 60 °C.

After cooling, the filament was passed through a drying unit and fed into the drawing section, consisting of two independently driven five-roll units. Orientation drawing was carried out in a hot water bath at 90 °C. This temperature was selected because it is above the glass transition temperature of PET, allowing the material to enter a highly elastic state with sufficient chain mobility for orientation, while remaining significantly below the cold crystallization region. Under these conditions, structural ordering and crystallization predominantly develop as a consequence of deformation-induced molecular alignment rather than purely thermal effects. In addition, the selected temperature enabled stable drawing and direct comparison of all investigated compositions under identical processing conditions.

The draw ratio (DR) was defined by the speed difference between the two roll units and was varied in the range of 1.0 to 6.5. Drawing was performed in an inline mode directly after extrusion. No post-drawing thermal fixation was applied.

After processing, the filaments were wound under minimal tension and conditioned at room temperature for 72 h prior to testing.

### 2.3. Mechanical and Physical Characterization

Mechanical properties were evaluated for both as-extruded monofilaments and injection-molded specimens. Injection molding was performed using a vertical hydraulic molding machine at a melt temperature of 265 °C, a mold temperature of 50 °C, and an injection pressure of 320 bar. Processing conditions were kept constant for all materials.

Tensile properties of monofilaments were measured in accordance with ISO 527-2 [28], while tensile modulus was determined using injection-molded specimens according to ISO 527-1 [29]. Density was measured using the immersion method in accordance with ISO 1183-1 [30].

Melt flow index (MFI) was determined according to ISO 1133 [31], under a load of 2.16 kg using a standard die (diameter 2.095 mm). All samples were dried prior to testing to ensure reproducible results.

Thermal shrinkage was evaluated by heat treatment in a forced-air oven at 100 °C for 2 h. Linear dimensional changes were determined according to ISO 16012 [32], after conditioning the samples at room temperature for 24 h prior to testing.

**Figure 2:**
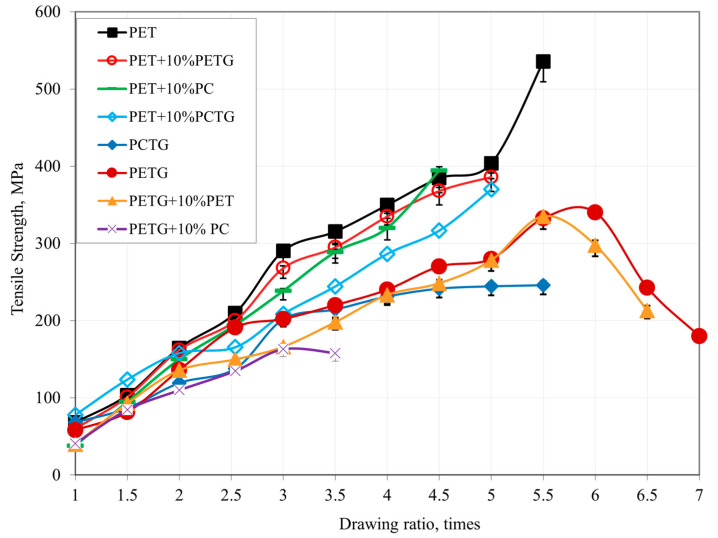
Tensile strength as a function of draw ratio for oriented filament samples.

**Figure 3:**
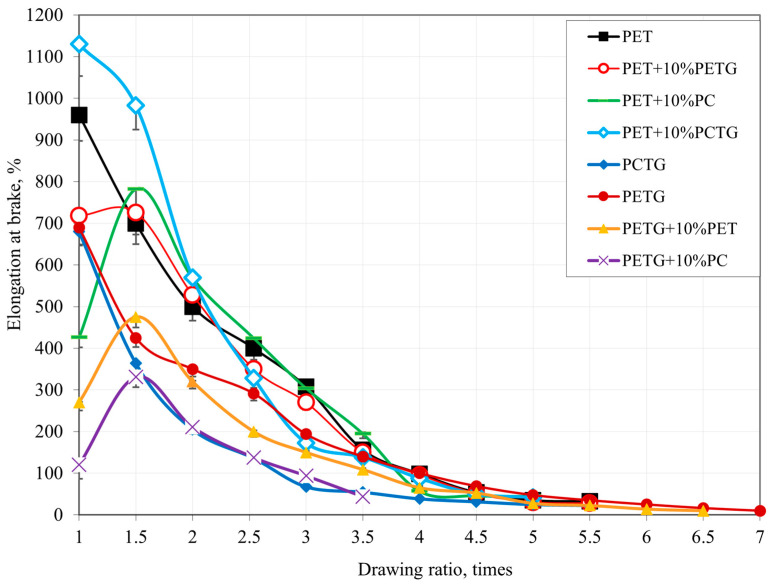
Elongation at break as a function of draw ratio for oriented filament samples.

**Figure 4:**
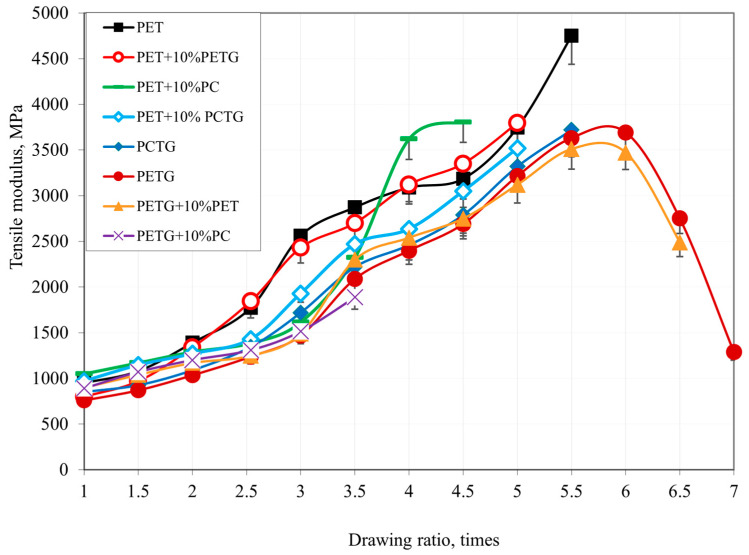
Tensile modulus as a function of draw ratio for oriented filament samples.

**Figure 5:**
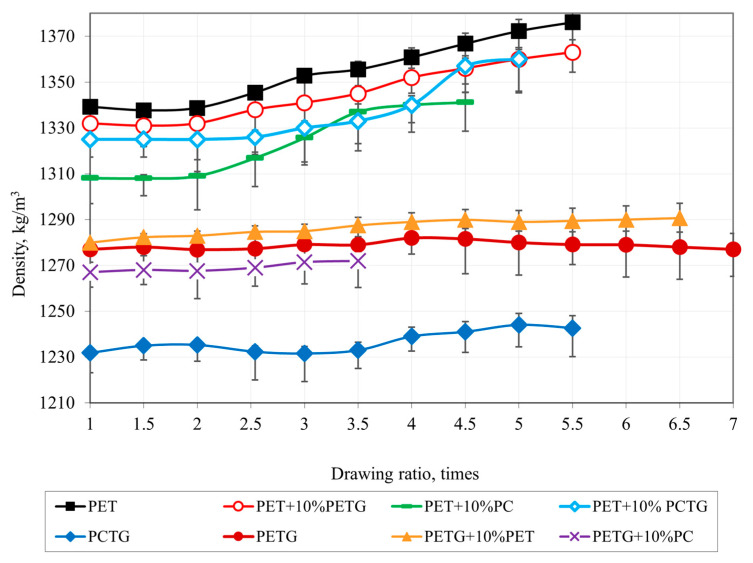
Density as a function of draw ratio for oriented filament samples.

**Figure 6:**
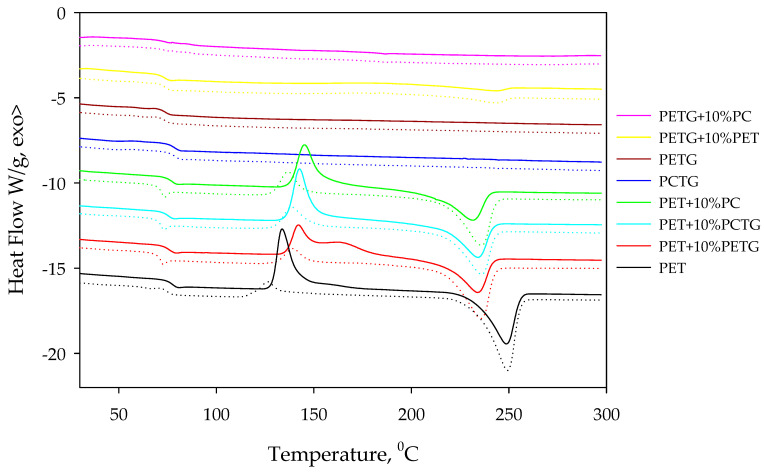
DSC thermograms of selected monofilament samples before drawing (solid lines, DR = 1) and after orientation drawing to the maximum achievable draw ratio (dashed lines).

All mechanical and physical measurements were performed using at least six replicates for each data point to ensure statistical reliability. The reported values represent the arithmetic mean, and the standard deviation was used to estimate experimental uncertainty and is shown as error bars in Figure 2, Figure 3, Figure 4, Figure 5 and Figure 6.

Differential scanning calorimetry (DSC) analysis was performed for selected monofilament samples in the initial as-extruded state (DR = 1) and after orientation drawing to the maximum achievable draw ratio. The selected samples represent the limiting structural states before and after orientation-induced structural development. Thermal behavior was analyzed using a DSC Q20 differential scanning calorimeter (TA Instruments, New York, NY, USA). Samples with a mass of approximately 7–8 mg were cut from the monofilaments and sealed in standard aluminum pans. Measurements were carried out during heating from 20 to 300 °C at a heating rate of 10 °C/min under a dry nitrogen atmosphere with a purge flow of 50 mL/min. The glass transition temperature (T_g_), cold crystallization temperature (T_cc_), melting temperature (T_m_), and corresponding enthalpies were determined from the thermograms. The degree of crystallinity was estimated using the melting enthalpy of 100% crystalline PET (ΔH_m100%_ = 140 J/g).

## 3. Results and Discussion

### 3.1. Initial Properties and Processability of Polymer Blends

The initial properties of the investigated materials and their blends are summarized in Table 2. A progressive increase in melt flow index (MFI) is observed after each processing step, indicating a reduction in molecular weight due to thermal and mechanical degradation during extrusion and reprocessing. This effect is particularly pronounced for blends containing polycarbonate, where a significant increase in MFI suggests possible intermolecular reactions, such as transesterification, leading to chain scission and reduced melt viscosity.

Mechanical properties of injection-molded samples indicate that the addition of PETG to PET has only a minor effect on tensile strength and modulus, suggesting a certain degree of compatibility between these components. In contrast, the incorporation of polycarbonate results in a noticeable decrease in tensile strength, which is likely associated with partial phase incompatibility, leading to less efficient stress transfer across phase boundaries.

A substantial difference is observed between injection-molded and as-extruded filament samples. Even at minimal take-up speeds, the extrusion process induces partial molecular orientation due to extensional flow in the die, resulting in higher tensile strength and elongation of filament samples. This highlights the sensitivity of polyester systems to processing-induced orientation effects.

The maximum achievable draw ratio varies significantly among the investigated systems and reflects their ability to undergo stable deformation during orientation drawing. PET and PET-rich blends exhibit draw ratios up to ~5.5, while PETG-containing systems reach values up to ~7, indicating higher deformability but not necessarily improved mechanical performance. In contrast, blends containing polycarbonate show reduced drawability (down to ~3.5–4.5), suggesting limited orientation capability and the early onset of structural instability.

### 3.2. Effect of Orientation Drawing on Tensile Strength and Elongation

For all materials, tensile strength increases with increasing draw ratio, which is associated with progressive molecular alignment along the drawing direction. During drawing, the initially isotropic entangled structure gradually transforms into an oriented load-bearing network capable of more efficient stress transfer. Chain segments become increasingly aligned, while intermolecular interactions and tie molecules contribute to improved load transfer efficiency within the oriented structure [11,12,33]. However, the extent of this increase strongly depends on the material composition.

Pure PET exhibits the highest tensile strength at all draw ratios. This behavior is consistent with the development of strain-induced crystallization (SIC) together with progressive reorganization of the initial lamellar structure during drawing [11,12]. Deformation involves alignment of amorphous chains, lamellar rotation, interlamellar slip, and partial fragmentation of the crystalline phase, leading to the development of a fibrillar morphology. These structural changes, along with an increasing fraction of load-bearing tie molecules, enhance stress transfer along the drawing direction [11,12].

PET/PETG blends show a similar but slightly reduced increase in tensile strength. The presence of the amorphous PETG phase modifies the structural evolution during drawing in several ways. Due to its irregular chain structure, PETG has a limited ability to crystallize and therefore reduces the extent of strain-induced crystallization in the system [10,23]. In addition, the PETG phase disrupts the continuity of the load-bearing structure by introducing mechanically softer amorphous regions that do not effectively carry load [13,14]. Furthermore, deformation of PET and PETG is not fully cooperative: while PET undergoes orientation and partial crystallization, PETG primarily deforms through chain alignment without contributing to structural reinforcement [23]. As a result, stress transfer becomes less efficient, leading to reduced tensile strength compared to pure PET.

In contrast, PETG and PETG-rich blends exhibit non-monotonic behavior, with a decrease in tensile strength at high draw ratios (>5.5). Since PETG is predominantly amorphous, its deformation is governed mainly by chain alignment without significant crystallization [13,23]. At moderate draw ratios, this leads to strengthening due to molecular alignment. However, in the absence of crystallization, no stable load-bearing structure is formed. At higher draw ratios, the oriented amorphous structure becomes less effective in sustaining stress, and deformation may involve chain slippage, disentanglement, and strain localization [13,23]. Structural heterogeneity can further promote localized deformation, resulting in premature weakening at high draw ratios.

Blends containing polycarbonate exhibit both lower tensile strength and a reduced maximum draw ratio. This behavior is associated with the presence of a high-glass-transition amorphous phase (PC), which remains in a glassy or near-glassy state under the applied drawing conditions and therefore does not effectively participate in orientation. As a result, deformation of PET and PC is strongly non-cooperative. While the PET phase undergoes alignment and structural reorganization, the PC phase behaves as a mechanically rigid inclusion. This behavior is likely associated with stress concentration at phase boundaries caused by limited interfacial adhesion, resulting in inefficient stress transfer between phases [13,14]. At higher draw ratios, these interfacial regions may act as sites of localized deformation or cavitation, resulting in reduced tensile strength and limited deformability [34].

Overall, the evolution of tensile behavior reflects competition between orientation-induced reinforcement and structural limitations imposed by amorphous or partially incompatible phases. Systems capable of developing strain-induced crystallization exhibit more efficient stabilization of the oriented structure, whereas predominantly amorphous systems rely mainly on entanglement-controlled deformation and therefore show reduced strengthening efficiency at high draw ratios [13,14,23].

The elongation at break as a function of draw ratio is shown in Figure 3. The initial elongation values (DR = 1) vary significantly among the investigated systems, indicating differences in their as-extruded structure. These differences arise from variations in residual orientation, phase morphology, and chain mobility. Systems such as PET and PET + 10% PCTG exhibit high elongation, suggesting a relatively homogeneous and less constrained structure, whereas blends containing PC or certain PETG-based compositions show lower elongation, which can be attributed to restricted chain mobility and structural heterogeneity.

A distinct feature is observed for several systems (PET + 10% PC, PETG + 10% PET, and PETG + 10% PC), which exhibit a pronounced increase in elongation at low draw ratios (DR ≈ 1.5). This behavior is attributed to the relaxation of residual stresses and partial release of frozen-in orientation introduced during extrusion. At low deformation levels, structural rearrangement and improved homogeneity enable more effective plastic deformation, resulting in a temporary increase in ductility.

With a further increase in draw ratio, all systems exhibit a monotonic decrease in elongation. This reduction is associated with progressive chain orientation and alignment, leading to decreased conformational freedom and increased structural anisotropy. As the structure becomes more oriented, deformation becomes localized, and the material fails at lower strains. In semicrystalline systems such as PET, this effect is further enhanced by strain-induced crystallization, which stabilizes the oriented structure and limits chain mobility. The observed reduction in elongation at break with increasing draw ratio is consistent with previous studies on oriented PET fibers and related polyester systems, where progressive molecular alignment and crystallization reduce segmental mobility and suppress large-scale plastic deformation at high orientation levels [11,12,24].

At high draw ratios (DR > 4), the elongation reaches a low plateau for all materials, indicating that the structure has transitioned into a highly oriented and mechanically constrained state. In this regime, the ability of the material to undergo further plastic deformation is strongly limited, regardless of composition.

Although direct morphological characterization was not performed in the present study, the observed behavior is consistent with morphology-related effects commonly reported for partially compatible polyester/polycarbonate systems [13,14,15,16,23].

### 3.3. Tensile Modulus Development

As shown in Figure 4, the tensile modulus increases with draw ratio for all investigated materials, reflecting progressive chain orientation and alignment along the drawing direction. This leads to reduced conformational freedom and the development of a stiffer, more load-bearing structure. The increase in modulus is associated not only with chain alignment itself but also with restricted segmental mobility and reduced conformational freedom in the oriented state. As orientation develops, deformation increasingly occurs along covalent backbone directions rather than through chain conformational rearrangements, resulting in enhanced stiffness [11,12].

Pure PET exhibits the highest modulus values over the entire range of draw ratios, reaching a maximum at DR ≈ 5–5.5. This behavior is associated with the combined effect of chain alignment, lamellar reorganization, and strain-induced crystallization, which stabilizes the oriented structure and enhances stiffness.

Blends containing PETG show slightly lower modulus values compared to PET. The presence of the amorphous PETG phase reduces the extent of crystallization and disrupts the continuity of the load-bearing structure. As a result, although orientation and alignment occur, the increase in stiffness is less pronounced than in pure PET.

At high draw ratios, PETG-rich systems exhibit a decrease in modulus. This behavior is not related to an inherent instability of the amorphous phase, but rather to the limited ability of the oriented amorphous structure to maintain efficient stress transfer at high levels of deformation. In the absence of strain-induced crystallization, the load-bearing network is governed primarily by entanglements, which may become less effective due to chain slippage, disentanglement, or partial relaxation of the oriented structure under the applied conditions [13,23].

Blends containing polycarbonate exhibit relatively high modulus values at moderate draw ratios (DR ≈ 4–5), which can be attributed to the intrinsically high stiffness of the PC phase. However, due to its high glass transition temperature, PC does not effectively participate in orientation during drawing. As a result, further increase in modulus at higher draw ratios is limited. In addition, non-cooperative deformation of PET and PC phases and restricted stress transfer across the interface prevent the development of a fully efficient oriented structure, thereby limiting the overall mechanical performance.

### 3.4. Density Evolution and Crystallization Behavior

The evolution of material density with draw ratio (Figure 5) provides insight into structural changes occurring during orientation drawing, particularly those related to molecular packing and the development of ordered regions.

PET and PET-rich blends exhibit a clear increase in density with increasing draw ratio. This behavior is consistent with progressive structural ordering during deformation, including molecular alignment, lamellar reorganization, and strain-induced crystallization. The increase in density reflects more efficient chain packing and the development of a more ordered structure [11,12].

In contrast, PETG, PCTG, and PETG-rich blends show only minor changes in density over the investigated draw ratio range. This indicates that structural evolution in these systems is dominated by chain alignment within the amorphous phase, while the formation of crystalline regions remains limited, which is typical for amorphous copolyesters with suppressed crystallization ability [10,23].

Blends such as PET + 10% PC exhibit intermediate behavior, with a moderate increase in density. This indicates that crystallization of the PET phase still develops during drawing but is partially restricted. This effect can be attributed not only to dilution of the crystallizable component but also to constraints imposed by the amorphous phase.

In partially miscible systems, the presence of an amorphous component may lead to the formation of an interphase region with reduced chain mobility [20]. In this region, segmental rearrangements required for crystallization are hindered, thereby limiting the extent of thermal crystallization. Under such conditions, structural ordering is promoted primarily through orientation-induced chain alignment and strain-induced crystallization during drawing.

Overall, the observed density evolution indicates that the ability to develop an ordered structure during orientation strongly depends on composition: PET exhibits the highest degree of structural development, while amorphous-rich systems remain largely limited to orientation without significant crystallization.

To further evaluate structural ordering during orientation drawing, DSC analysis was performed for selected monofilament samples in the initial state and after drawing to the maximum achievable draw ratio. The obtained thermal parameters are summarized in Table 3, while representative thermograms are shown in Figure 6.

PET and PET-rich blends exhibited a pronounced increase in melting enthalpy and crystallinity after orientation drawing. Simultaneously, the cold crystallization enthalpy decreased significantly, while the cold crystallization temperature shifted to lower values after drawing. This behavior indicates that partial structural ordering and nucleation develop during deformation prior to DSC heating, facilitating crystallization during subsequent heating scans. Such behavior is characteristic of strain-induced crystallization in oriented PET systems [11,12]. For PET-containing systems, a slight decrease in glass transition temperature was observed after drawing. This behavior may be associated with partial relaxation and nonequilibrium packing within the residual amorphous phase formed during orientation drawing [11,12,23].

Among the investigated blends, PET + 10% PETG and PET + 10% PCTG exhibited similar trends but with lower crystallinity values compared to neat PET. This behavior confirms that the amorphous copolyester phases partially suppress crystallization development and reduce the efficiency of strain-induced crystallization. The suppression effect was more pronounced for the PCTG-containing blend, which exhibited lower crystallinity and higher residual cold crystallization enthalpy after drawing, indicating more restricted structural ordering during deformation. In addition, PET-containing blends exhibited a slight increase in melting temperature after drawing, which may be associated with the formation of more stable and better organized crystalline regions during orientation-induced crystallization.

In the PET + 10% PC system, crystallinity development was also limited compared to neat PET. This behavior is consistent with restricted chain mobility and non-cooperative deformation of the PET and PC phases, which reduces the ability of the oriented structure to reorganize into crystalline regions during drawing.

In contrast, PETG and PCTG samples remained predominantly amorphous and exhibited only glass transition behavior without detectable melting peaks even after orientation drawing. In these fully amorphous systems, the glass transition temperature remained essentially unchanged after drawing, indicating that orientation development occurs mainly through chain alignment within the amorphous phase without substantial crystallization or rigid phase formation [13,23]. A similar behavior was observed for the PETG + 10% PC blend, which also remained predominantly amorphous after orientation drawing. The PETG + 10% PET blend exhibited weak crystallization and melting behavior with low crystallinity values, indicating limited but detectable crystallization of the PET phase within the predominantly amorphous PETG matrix [10,23].

Overall, the DSC results support the interpretation of density evolution and confirm that the increase in density observed in PET-containing systems is associated with crystallization development during orientation drawing rather than solely with densification or packing rearrangement within the amorphous phase.

### 3.5. Thermal Shrinkage and Structural Stability

**Figure 7:**
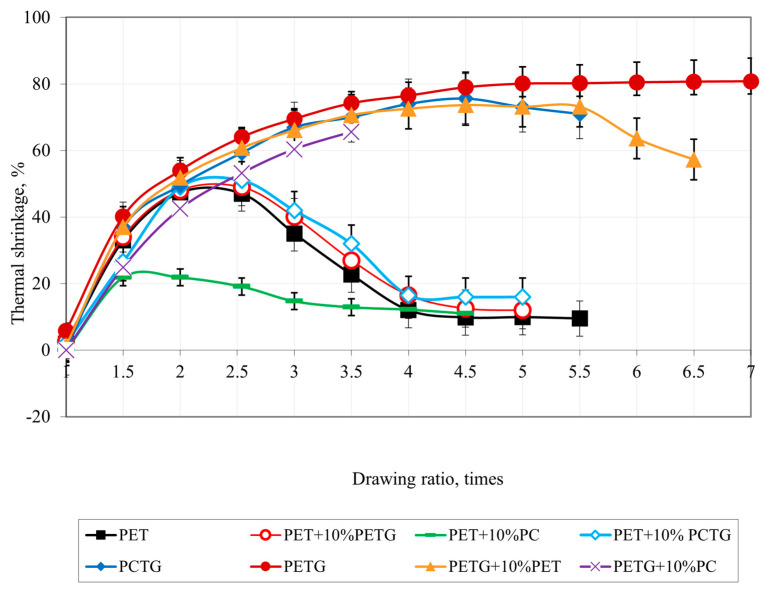
Thermal shrinkage as a function of draw ratio for oriented filament samples.

Thermal shrinkage results (Figure 7) reveal significant differences in the stability of the oriented structure among the investigated systems. Thermal shrinkage reflects the extent to which the oriented structure relaxes upon heating and is therefore closely related to the mechanisms responsible for stabilizing molecular orientation. Thermal shrinkage behavior reflects the balance between stored molecular orientation and the ability of the structure to stabilize the oriented state through crystallization. In semicrystalline systems, crystalline regions act as physical constraints that suppress chain relaxation during heating, whereas predominantly amorphous systems exhibit greater structural recovery due to increased segmental mobility [11,12,23].

PET and PET-based blends exhibit relatively low shrinkage values (down to ~10% at high draw ratios), indicating effective stabilization of the oriented structure. This behavior is primarily associated with strain-induced crystallization, which anchors molecular chains and restricts their ability to relax during heating [11,12].

In contrast, PETG, PCTG, and their blends show significantly higher shrinkage values. In these systems, orientation is mainly stored within the amorphous phase, and in the absence of crystallization, there are no stable structural elements to fix the aligned chains. Upon heating, increased chain mobility leads to relaxation of the oriented state, resulting in substantial dimensional shrinkage.

Blends containing polycarbonate exhibit relatively low shrinkage at low to moderate draw ratios. This behavior can be attributed to the high glass transition temperature of the PC phase, which limits chain mobility under the applied conditions and suppresses relaxation of the oriented structure. However, this effect does not necessarily indicate efficient structural stabilization, but rather reflects restricted molecular mobility.

### 3.6. Structure–Property Relationships

Overall, the mechanical performance of oriented polyester systems is governed by the balance between chain alignment, crystallization, and the ability to maintain an effective load-bearing structure during deformation and subsequent thermal exposure. Semi-crystalline PET exhibits superior performance due to strain-induced crystallization, which stabilizes the oriented structure and enhances stress transfer. The incorporation of amorphous copolyesters (PETG, PCTG) reduces the extent of crystallization and shifts the deformation mechanism toward alignment within the amorphous phase, resulting in lower stiffness and reduced structural stability. In contrast, polycarbonate-containing blends are characterized by limited chain mobility and non-cooperative deformation, which restrict orientation and reduce strength, despite contributing to stiffness at moderate draw ratios. These results demonstrate that even small compositional variations significantly affect structural development, drawability, and the balance between strength, stiffness, and dimensional stability.

## 4. Conclusions

The present study demonstrates that the orientation behavior and resulting mechanical performance of PET-based systems are governed by the combined effects of chain alignment, crystallization, and structural stabilization during deformation.

Semi-crystalline PET exhibits superior tensile strength and modulus due to its ability to undergo strain-induced crystallization, which stabilizes the oriented structure and reduces thermal shrinkage. In contrast, amorphous copolyesters such as PETG and PCTG limit crystallization and shift the deformation mechanism toward chain alignment within the amorphous phase, resulting in lower stiffness and increased shrinkage.

The incorporation of amorphous components into PET significantly alters structural development during drawing, even at relatively low concentrations (~10 wt%), reducing orientation efficiency and dimensional stability.

Blends containing polycarbonate exhibit reduced drawability and tensile strength due to limited chain mobility and non-cooperative deformation. The relatively low thermal shrinkage observed in these systems is associated with restricted molecular mobility rather than effective stabilization of the oriented structure.

Overall, the results establish clear structure–property relationships in oriented polyester blends and demonstrate that minor compositional variations can significantly affect processing stability and final material performance.

These findings highlight the importance of feedstock control and polymer separation in recycling processes targeting high-performance applications such as strapping tapes and monofilaments. In addition, the sensitivity of drawability and mechanical response to small compositional changes suggests that orientation drawing may serve as a practical indirect indicator of material composition in recycled PET streams.

## Figures and Tables

**Table 1:** Composition of investigated systems (wt%).

Component	PET	PETG	PET + 10% PETG	PETG + 10% PET	PET + 10% PC	PETG + 10% PC	PCTG	PET + 10% PCTG
PET	100	0	90	10	90	0	0	90
PETG	0	100	10	90	0	90	0	0
PC	0	0	0	0	10	10	0	0
PCTG	0	0	0	0	0	0	100	10

**Table 2:** Initial properties of the different material samples.

Properties	Blend								
	PET	PETG	PET + 10% PETG	PETG + 10% PET	PET + 10% PC	PETG + 10% PC	PCTG	PC	PET + 10% PCTG
	Granulated blends and initial materials								
MFI, g\10 min 265 °C	12	24	15	23	12	22	17	8	13
MFI, g\10 min 275 °C	18	33	23	33	19	29	24	11	21
	Injection-molded sample								
Tensile Strength, (MPa)	62	63	62	58	48	42	65	68	67
Elongation at break, %	240	450	320	280	170	210	250	110	230
Tensile modulus, MPa	1100	980	1000	1050	1200	1100	800	2100	900
MFI, g\10 min 265 °C	15	29	16	27	18	29	19	11	14
MFI, g\10 min 275 °C	23	38	27	38	27	37	26	14	23
	Monofilament sample without drawing								
Tensile Strength, (MPa)	68	58	60	40	38	41	68	72	78
Elongation at break, %	960	690	718	270	426	120	680	180	1130
Tensile modulus, MPa	950	760	800	900	1050	890	850	1700	970
Maximum draw ratio, times	5.5	7	5.5	6.5	4.5	3.5	5.5	-	5.0
MFI, g\10 min 265 °C	16	30	18	31	25	33	21	-	17
MFI, g\10 min 275 °C	26	40	32	43	48	49	28	-	27

**Table 3:** DSC thermal characteristics of selected monofilament samples before and after orientation drawing.

Sample	Draw Ratio (DR)	Tg (°C)	Tcc (°C)	ΔHcc (J/g)	Tm (°C)	ΔHm (J/g)	Crystallinity Xc (%)
PET	1.0 5.5	78 76	133 127	31.49 4.16	248 249	37.76 53.01	2 35
PET + 10% PETG	1.0 5.5	77 73	142 139	26.86 8.20	233 235	31.81 45.12	4 29
PET + 10% PC	1.0 4.5	78 74	145 137	23.87 12.08	232 235	26.51 37.15	2 20
PET + 10% PCTG	1.0 5.0	75 73	143 139	25.26 13.50	234 236	30.75 36.18	4 18
PETG + 10% PC	1.0 3.5	74, 140 74, 140	- -	- -	- -	- -	~0 ~0
PETG + 10% PET	1.0 6.5	76 74	178 178	2.26 2.04	243 243	2.99 4.30	7 16
PETG	1.0 7.0	74 74	- -	- -	- -	- -	~0 ~0
PCTG	1.0 5.5	79 79	- -	- -	- -	- -	~0 ~0

## Data Availability

The original contributions presented in this study are included in the article. Further inquiries can be directed to the corresponding author.

## Abbreviations

The following abbreviations are used in this manuscript:

PET	Polyethylene terephthalate
PETG	Polyethylene terephthalate glycol-modified
PCTG	Polycyclohexylenedimethylene terephthalate glycol-modified
PC	Polycarbonate
DR	Draw ratio
